# WALK 2.0 - Using Web 2.0 applications to promote health-related physical activity: A randomised controlled trial protocol

**DOI:** 10.1186/1471-2458-13-436

**Published:** 2013-05-03

**Authors:** Gregory S Kolt, Richard R Rosenkranz, Trevor N Savage, Anthony J Maeder, Corneel Vandelanotte, Mitch J Duncan, Cristina M Caperchione, Rhys Tague, Cindy Hooker, W Kerry Mummery

**Affiliations:** 1 University of Western Sydney, School of Science and Health, Sydney, NSW, Australia; 2 Department of Human Nutrition, Kansas State University, Manhattan, KS, USA; 3 University of Western Sydney, School of Computing, Engineering and Mathematics, Sydney, NSW, Australia; 4 Central Queensland University, Institute for Health and Social Science Research, Rockhampton, Queensland, Australia; 5 University of British Columbia, School of Health and Exercise Sciences, Kelowna, British Columbia, Canada; 6 University of Alberta, Faculty of Physical Education and Recreation, Edmonton, Alberta, Canada

**Keywords:** Physical activity, Randomised controlled trial, Internet

## Abstract

**Background:**

Physical inactivity is one of the leading modifiable causes of death and disease in Australia. National surveys indicate less than half of the Australian adult population are sufficiently active to obtain health benefits. The Internet is a potentially important medium for successfully communicating health messages to the general population and enabling individual behaviour change. Internet-based interventions have proven efficacy; however, intervention studies describing website usage objectively have reported a strong decline in usage, and high attrition rate, over the course of the interventions. Web 2.0 applications give users control over web content generated and present innovative possibilities to improve user engagement. There is, however, a need to assess the effectiveness of these applications in the general population. The Walk 2.0 project is a 3-arm randomised controlled trial investigating the effects of “next generation” web-based applications on engagement, retention, and subsequent physical activity behaviour change.

**Methods/design:**

504 individuals will be recruited from two sites in Australia, randomly allocated to one of two web-based interventions (Web 1.0 or Web 2.0) or a control group, and provided with a pedometer to monitor physical activity. The Web 1.0 intervention will provide participants with access to an existing physical activity website with limited interactivity. The Web 2.0 intervention will provide access to a website featuring Web 2.0 content, including social networking, blogs, and virtual walking groups. Control participants will receive a logbook to record their steps. All groups will receive similar educational material on setting goals and increasing physical activity. The primary outcomes are objectively measured physical activity and website engagement and retention. Other outcomes measured include quality of life, psychosocial correlates, and anthropometric measurements. Outcomes will be measured at baseline, 3, 12 and 18 months.

**Discussion:**

The findings of this study will provide increased understanding of the benefit of new web-based technologies and applications in engaging and retaining participants on web-based intervention sites, with the aim of improved health behaviour change outcomes.

**Trial registration:**

Australian New Zealand Clinical Trials Registry, ACTRN12611000157976

## Background

Physical inactivity remains one of the leading modifiable causes of death and disease in Australia [1]. Regular physical activity (PA) decreases the risk of developing cardiovascular disease, diabetes, some cancers, obesity, osteoporosis, and other chronic conditions [2], but national Australian surveys indicate that almost 60% of Australians aged 15 years and over do not undertake sufficient PA to obtain health benefits [3]. It has been estimated that physical inactivity contributes to more than 8,000 deaths in Australia each year, and that for every 1% increase in the Australian population becoming sufficiently physically active some $7.2 million in health care costs could be saved [4]. Novel approaches for increasing PA with the potential to reach broad populations at an acceptable cost are needed.

Interventions delivered via the Internet have emerged as a novel and popular health promotion strategy, with the potential for wide population reach. The Internet is potentially an important medium in communicating messages associated with raising the profile of PA to the general population [5,6]. Over the past decade, there has been unprecedented growth in the use of the Internet world-wide [7,8]. For example, in Australia (the focus of this trial) in 2010–2011 79% of homes had Internet access and there were over 6.2 million households with broadband Internet access (73% of all homes), showing a five-fold increase over the past ten years in Australia [9] and six-fold increase worldwide [7]. Internet users are also becoming more representative of the overall population, as more women, elderly, and people from low socio-economic background are going online [10]. The exponential growth of the Internet has been paralleled by research into the uses of the Internet for social marketing and health promotion [11]. Recent reviews of the effectiveness of Internet-based PA interventions have also demonstrated the short-term efficacy of this medium for individual behavioural change, but also the need to increase user interaction and retention to websites to increase long-term behavioural outcomes [12-15].

To obtain the social marketing, health promotion, and behavioural change benefits associated with Internet-delivered PA interventions, the engagement and retention of participants in larger and more representative study samples must be addressed [15]. Intervention studies that have provided objective data on website usage have reported a strong decline in usage, and high attrition rate over the course of the intervention [15]. A low level of website interactivity has been suggested as an explanation for the modest retention and engagement rates within these health promotion trials [16]. There is consensus in the literature that website-delivered behavioural change interventions with a high degree of interactivity are more effective in producing behaviour change compared to those with a low degree of interactivity [17,18].

Web 2.0 represents the newest generation of Internet-based, highly interactive applications, which are aimed at giving users control of how information is generated, created, and shared. Web 2.0 applications, including blogs, wikis, podcasts, mash-ups, and social networking sites, are widely embraced and their popularity continues to grow. For example, in Australia, there are currently over 11 million people who use the social networking website Facebook, with 75% of users going to the site at least once a day [19]. Hence, there are a growing number of speculative theses on the potential of Web 2.0 applications in the fields of health and medicine [20-24]. There is a clear need for larger population studies to examine this next generation of web-based applications (Web 2.0) and to study their effectiveness relative to conventional web-based approaches (Web 1.0), particularly with regard to participant engagement, retention, and PA behaviour change.

The 10,000 Steps program is one example of a novel PA promotion project that was initially established as a whole-of-community study known as the 10,000 Steps Rockhampton project [25,26]. Since the completion of the original project, the program has continued to be disseminated at the individual and community levels [5]. A key element of the dissemination of the program has been the use of the Internet to promote and support PA. The 10,000 Steps website has been used to disseminate PA information to health professionals and to provide engagement of registered members through the use of an online step log. The website is generally used in conjunction with a pedometer, one of the novel elements of the original program, and web-based ‘i-challenges’ and ‘virtual journeys’. The 10,000 Steps website has demonstrated that the self-monitoring characteristic of the site (use of i-challenges) is a strong factor in retaining users [27,28]. This is consistent with research demonstrating that pedometers are useful as self-monitoring and motivational tools, and also a significant factor in increasing PA in a broad range of population groups including people with type 2 diabetes [29], insufficiently active women [30], and people engaged in a web-based worksite PA program [31]. Pedometer step logs, in conjunction with activities such as challenges and ‘virtual journeys’, are basic examples of the type of interactivity that can be created using Web 1.0 technology, which may further enhance the capacity of websites to retain users and produce more sustainable changes in PA.

In this study we plan to compare the use of Web 1.0 applications, as applied in the Australian 10,000 Steps website, with Web 2.0 applications. Our aim is to investigate the effects of “next generation” web-based applications (e.g. social networking), to establish whether Web 2.0 technology can outperform Web 1.0 technology in terms of website engagement, retention and subsequent PA behaviour change.

Primary Hypothesis:

H1: Participants in the Web 2.0 condition will display higher levels of physical activity at 3 months, and at 12 and 18 months post intervention, compared with the Web 1.0 or control condition.

Secondary Hypothesis:

H2: There will be significantly greater website engagement and participant retention on the website in the Web 2.0 arm of the study than in the conventional (Web 1.0) condition at 3 months, and at 12 and 18 months post intervention.

## Methods/design

### Trial design

**Figure 1:**
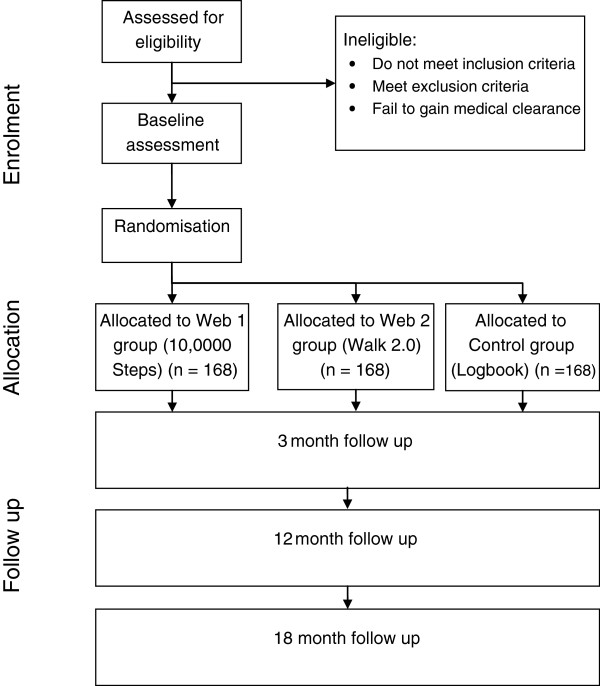
Flow diagram of study protocol.

The Walk 2.0 project is a three-arm randomised controlled trial (RCT) investigating the effectiveness of two web-based PA interventions and a logbook PA intervention (Figure 1). Outcomes will be assessed at baseline, and at 3, 12, and 18 months. The study has received ethics approval from the Human Research Ethics Committees of the University of Western Sydney (Reference number H8767) and CQUniversity (H11/01-005). The study will be reported according to CONSORT guidelines [32].

### Participants and recruitment

A total of 504 participants will be recruited for this study across two sites in Australia (South Western Sydney and Central Queensland). The primary means of recruiting participants will be a personalised invitation letter to an extract of randomly selected individuals from the Australian Electoral Commission (AEC) electoral roll. Voting is compulsory for persons aged 18 years and over in Australia and AEC data provides an effective means of sampling the targeted population. Previous research utilising a similar method to recruit participants, but requiring mainly survey-based cohort measurements, reported that between 60 and 70% of individuals invited to a research project using this manner, participated [28,29]. The participant requirements of this research, however, are higher than these other studies, and, thus a response rate of less than 10% is anticipated. Therefore, an extract of 14,000 names and addresses - 7,000 from each of the Federal electoral divisions of Capricornia (Rockhampton, QLD) and Werriwa (South Western Sydney, NSW) - matched by age and gender, was obtained from the AEC electoral roll. A number of other recruitment methods will also be utilised to supplement the primary recruitment method and to recruit a diverse range of participants. These methods include advertising in local print media, calling former research participants who had registered their interest in participating in future research, and messages delivered through university email lists.

### Eligibility

To be eligible for the study, individuals must live or work in South Western Sydney (New South Wales) or Rockhampton (Queensland), be willing to increase the amount of physical activity that they are currently taking part in, and be over 18 years of age. Participants will be excluded from the project if:

1. They do not have access to the Internet.

2. Are unable to speak/read English.

3. They are currently engaging in moderate-to-vigorous PA (MVPA) for 30 minutes on 5 or more days per week, with the question “As a rule, do you do at least half an hour of moderate or vigorous exercise (such as walking or a sport) on five or more days a week?” [33].

4. They have an existing medical condition which could be exacerbated by PA (assessed using the Physical Activity Readiness Questionnaire, PAR-Q [34]).

5. They have ever been a member of http://www.10000steps.org.au.

Subsequent to the commencement of recruitment, additional screening for participants who self-identified as being either too physically active to participate in the study or as having an existing chronic medical condition (based on the PAR-Q measure) was introduced and will be conducted by telephone interview. The Active Australia questionnaire will be used to address potential recall bias by confirming the participant’s assessment of PA [35,36]. Individuals who self-identify as having an existing chronic medical condition will be invited to gain medical clearance from their general medical practitioner (family physician) to participate in the study, as some people are likely to still benefit from participating in the study given suitable medical clearance (e.g., those taking prescribed blood pressure medications).

### Study procedure

Participant eligibility will be screened through a self-administered survey that is delivered with a personalised invitation letter (AEC-recruited participants) and a reply-paid envelope. Online access to the screening survey will also be available for all potential participants. As indicated above, additional screening concerning PA and health status may be needed for some people. All those deemed eligible will then be contacted by telephone and invited to attend an induction session to provide informed consent and be fitted with an ActiGraph activity monitor to measure PA, which they are required to wear for 7 days. Participants will be asked to complete a log of wear time, showing time that the monitor was put on and taken off each day and the time and reason that the monitor was taken off during the day. When participants return the ActiGraph (at least 8 days later) they will participate in the baseline measurement session. All measurements sessions (baseline, 3, 12 and 18 months) will incorporate anthropometic measures and a self report questionnaire (Table 1) and will be conducted at a University campus. ActiGraph monitors will be posted to participants by registered post for 3, 12 and 18 month follow ups a week before they attend their measurement session at the university. Participants will be randomly allocated to an intervention condition and all participants will receive a pedometer (Yamax Digiwalker SW200, Yamasa Tokei Keiki co., Japan) after satisfactorily completing the requirements of the baseline measurement (valid Actigraph data, completed anthropometric assessments, completed baseline survey). Participants’ progress and use of the website will not be monitored between follow-up measurement points.

**Table 1:** Summary of measures to be collected

**Primary outcome measures**	**Data collection instrument**	**Collection points (months)**
Physical activity levels	7 day ActiGraph physical activity monitoring	0, 3, 12 and 18
	Active Australia Survey [35]	0, 3, 12 and 18
**Secondary outcome measures**		
Anthropometric measurements	Height	Induction, 0, 3, 12 and 18
	Weight	Induction, 0, 3, 12 and 18
	Abdomen girth [37]	Induction, 0, 3, 12 and 18
**Other measures**		
Self reported quality of life	SF-36 [38]	0, 3, 12 and 18
Psychosocial Correlates	Stages of Change [39]	0, 3, 12 and 18
	Intention [40]	
	Subjective Norm [40]	
	Perceived Behavioural Control [40]	
	Attitude [40]	
	Outcome expectations [41]	
	Self Efficacy [42]	
	Barriers Self Efficacy [42]	
Self reported Internet self-efficacy	Internet self-efficacy scale [43]	0, 3, 12 and 18
User satisfaction	System Usability Scale [44]	3, 12 and 18 months
Descriptive information	Demographics questionnaire	0

If valid Actigraph data are not collected at a time point, the participant will be asked to wear the ActiGraph activity monitor for a further 7 days until valid data have been collected. Anthropometric measurements will be collected at all time points. If participants miss an assessment point at 3 or 12 months, they will still be invited to attend the next subsequent follow-up assessment session.

### Interventions

#### Web 1.0 group

Participants in the Web 1.0 condition will gain access to the existing 10,000 Steps website (http://www.10000steps.org.au). The 10,000 Steps website is designed in conjunction with the use of a pedometer and offers a step log and individual self-monitoring features as well as numerous written (electronic) educational/informational materials [27]. These features allow the core functionality of recording steps and monitoring progress over time in a web-based environment. Communication between participants on this site is limited to a forum and a virtual walking buddy feature which enables a user to share their step log with another user. In order to request a walking buddy, users must know the email address of their potential walking buddy and invite them via email; alternatively users can post their email address on the public forum and ask to be invited as a walking buddy by interested individuals. There is no function on the 10,000 Steps website that allows users to search for other users.

#### Web 2.0 group

Participants in the Web 2.0 condition will have access to a newly developed website (Walk 2.0) featuring Web 2.0 features The Walk 2.0 website has been developed to replicate the core functionality of the 10,000 Steps website with additional Web 2.0 features. These additional features have been developed around blogs, Google Mash-ups, social networking, and other Web 2.0 architecture. The website, when used with a pedometer, allows core functionality of recording steps and monitoring progress over time in a web-based environment while facilitating contact between participants through ‘status updates’, streams, blogs, virtual walking groups, internal emails, and forum posts. Participants have their own home page, allowing them to access specific information about their progress and personalised features for the site, such as mapping their favourite walks using a Google ‘mashup’ tool, ‘friend’ other users, access their friend’s content (providing consent has been obtained), and invite outside friends and family, who will be able to use the website but not be involved in the trial, to join the site. Users also have a profile page which allows them to share selected information with their ‘friends’ on the site. Access to the Web 2.0 platform will be restricted to those in the Web 2.0 arm and other individuals that participants invite to join them on the site.

#### Control group

Participants in the Control condition will have access to a paper-based log book and will be directed not to register or use the publicly available 10,000 Steps website. The log book provides participants with an overview of the key messages available through the other interventions, such as instruction in goal setting and increasing opportunities for PA and health gain, and a means of recording steps and monitoring progress over time. Each log book covers a period of 3 months and participants will receive enough log books at each follow-up measurement point to sustain their involvement in the project until the following measurement.

### Randomisation

Participants will be randomly assigned to one of the three trial arms using equal groups random allocation performed through a computer-generated algorithm. To avoid contamination in cases where participants reside in the same household, the first participant will be randomly assigned to a trial arm and the other participants from that household will also be allocated to the same trial arm. Randomisation occurs after the participant has completed all baseline measurements. Participants will receive a standardised introduction to the features of the intervention that they have been assigned to, which will include the use of the pedometer, self-monitoring, and setting goals in all groups; and how to modify account settings and access important features such as progress graphs and charts, forums, and the use of social media for the web-based groups.

### Outcome measurements

#### Physical activity

Physical activity will be evaluated using both subjective (Active Australia Survey) and objective (ActiGraph activity monitor) methods. The Active Australia Survey assesses both frequency and duration of walking for transport and recreation and MVPA [35]. The Active Australia Survey has established acceptable test-retest reliability and validity in the Australian adult population, and has been documented as a useful evaluative tool for detecting intervention related change in PA behaviours [36,45,46]. The ActiGraph activity monitor (ActiGraph GT3X, http://www.theActiGraph.com) will be used to objectively measure minutes of MVPA. The GT3X will be affixed to an elastic belt and worn on the waist. The validity and reliability of the GTX3 has been shown to be similar to the Actigraph GT1M devices in laboratory testing and for the measurement of everyday activities [47,48]. The reliability and validity of GT1M compared to other commercially available activity monitors has been previously established as [49].

During the induction session participants will be instructed on correct wear and fitting of the ActiGraph activity monitor. Participants will be asked to wear the unit over their right hip at the point of the anterior superior iliac spine, and fastened with the supplied elastic waist band. Participants will also be asked to complete an activity monitor log detailing times the monitor was removed and activities undertaken when the Actigraph was not worn. The Activity monitor will be worn for 7 full days during waking hours, except when swimming or bathing and participating in contact sports. Triaxial data are collected in 1 second epochs along with step counts and inclinometry. When participants attend their baseline appointment at least 8 days later, the activity monitor data will be inspected. For the purposes of this study, valid wear time will be determined as at least 600 minutes wear time per day on 5 days. Wear time will be evaluated using the criteria of 60 minutes of consecutive zero data and a 2 minute spike tolerance [50]. Participants with invalid data will be asked to wear the activity monitor for a further 7 days. If, for the baseline measurement, a participant refuses or if they agree but return invalid data up to three times, they will be excluded from the study but allowed to continue using the website. Participants with valid data will then complete a baseline survey before being randomised to their trial intervention.

For 3, 12 and 18 month outcome measurements, activity monitors and wear log sheets will be delivered to participants using registered post. Receipt of the activity monitor will be confirmed by telephone and participants will also be contacted by telephone 2–3 days prior to the appointment to check wear compliance and confirm the scheduled follow-up appointment. If the participant reports compliance with expected wear of the activity monitor they will then attend the University where wear time will be validated according to the protocol described at baseline. If the participant reports non-compliance with the expected wear of the activity monitor, the monitor will be left with them for a minimum of a further 5 days, during which time they will be expected to wear the monitor to comply with project requirements. If the participant chooses not to wear the ActiGraph again they will be asked to complete the outcome survey and will not be excluded from the project.

#### Anthropometric measurements

Height, weight, and abdomen girth will be measured at all outcome measurement sessions by project staff. Weight and height will be measured with the participant standing normally, with feet together and head in the Frankfurt plane, using Seca 700 mechanical balance scales and a Seca 220 measuring rod (Seca GmbH, Hamburg). Participants will be asked to remove their shoes and any heavy personal items/items of clothing for the measurement. Abdomen girth will be measured from as the ellipse projected on the transverse plane using the bilateral iliac crests as antipodal tangents using the Seca 203 measurement tape in accordance with the NIH protocol [37].

#### Other measures

Website usage, engagement, and retention for the Web 1.0 and Web 2.0 intervention groups will be measured using a commonly available web traffic analysis platform (Google analytics) and monitoring of user-generated content. Features monitored will include:

• Number of logins

• Frequency of step log use (and steps logged)

• Use of site stream (status updates, and comments on ‘friends’ stream)

• Page visits

• Goals set

• Time on website

• Number of friends (Web 2.0 only)

• Number of blog posts

In the Control group, logbooks will also be collected to enable comparison in use of common features (e.g., step log, goal setting) between interventions.

Quality of life will be assessed with the RAND 36 item Short Form Health Survey (SF-36) which evaluates 8 health concepts including limitations in physical activities and usual role activities because of health problems, bodily pain, general mental health, and vitality (energy and fatigue). The SF-36 has been validated on Australian populations [51], has demonstrated suitability for use in the general population [52,53], and is associated with the stage of motivational readiness to changes in physical activity [54,55].

Participants’ confidence in their ability to execute tasks and trouble shoot problems with the Internet will be measured using the Internet Self-Efficacy Scale (ISES). The effect of Internet self-efficacy is poorly understood in terms of Internet-delivered PA programs. The ISES uses 8 items assessed on a 7-point likert scale to assess a user’s understanding of Internet hardware and software, confidence in gathering information using the Internet and learning skills to use Internet programs, and ability to troubleshoot and resolve Internet problems. The ISES has shown good reliability and internal consistency [43].

A brief psychosocial questionnaire was used to assess key variables pertinent to follow-up analyses of intervention mediation and moderation. These variables include core constructs from the Transtheoretical Model (TTM [56]), Social Cognitive Theory (SCT [41]), and Theory of Planned Behavior (TPB [40]), and have been constructed to align with the target behaviour of taking 10,000 steps per day. From the TTM, Stages of Change is assessed by whether participants are currently taking 10,000 steps per day; if so how long they have been doing so; if not, whether they intend to, or are preparing or beginning to take 10,000 steps per day. This 6-item Stages of Change measure was modelled on a previously published scale [39]. From SCT, we developed a specific set of 4 items to assess self-efficacy for taking 10,000 steps per day, as well as a set of 10 items to assess self-efficacy to overcome common barriers to taking steps from existing guidelines [42]. A set of 9 items was used collectively to assess agreement with the SCT construct of outcome expectations associated with taking 10,000 steps per day. From TPB, we assessed intention with 2 items, subjective norm with 2 items, perceived behavioural control with 2 items, and attitude with 4 items, all with regard to being physically active at a level of taking 10,000 steps per day. These TPB constructs were tailored to the target behaviour of 10,000 steps per day, based on a set of previously published items [57].

Overall user satisfaction of the assigned intervention will be investigated at all follow-up time points using the System Usability Scale [44].

### Statistical power and sample size

Sample size for the RCT is based on the primary outcome measure, minutes of MVPA, as measured by the ActiGraph accelerometer. A review of web-based PA interventions suggests that studies which do not include aspects of Web 2.0 had a small effect on change in PA status of participants and had a dropout rate of approximately 40% [15]. Given the enhanced effects of the Web 2.0 intervention expected on the PA status of participants, the current study will be powered to detect a small to moderate change in minutes of MVPA. Therefore, to achieve 80% power to detect a small to moderate difference in PA between groups (control/Web 1.0/Web 2.0) at any given time point, approximately 120 participants per group will be required using an alpha level of 0.05. The number of participants per group has been inflated by 40% (n=168/group) to account for participant drop out while retaining adequate power to achieve study aims at 18 months.

### Statistical analysis

All analyses will follow intention to treat principles. Main comparison between groups will be performed using general linear mixed modelling. The impact of missing data will be addressed using multiple imputation. The sample size is inflated to incorporate the additional uncertainty arising from missing data. All analyses will be conducted using SPSS for Windows (Version 15.0). The level of significance (alpha) will be set at 0.05.

## Discussion

The current trial presents a unique opportunity to study the effectiveness of new generation web based applications (Web 2.0) in social marketing and health promotion using a conventional (Web 1.0), established, and ongoing PA promotion program (10,000 Steps Australia) as a comparator. Each year there is a proliferation of health-related and health promotion websites, yet very little work has been carried out to study the utility and effectiveness of these population-targeted websites [58]. Research studying the effectiveness of this now pervasive communication medium has been limited to studies of short duration and at times modestly sized samples [15], with many lacking any comparative study of website components [14]. We expect that the current study’s findings will provide increased understanding of the benefits of new web-based technologies and applications in engaging and retaining participants on web-based intervention sites, with the aim of improved and maintained health outcomes as a result of increased website engagement and retention.

## Abbreviations

AEC: Australian Electoral Commission; ISES: Internet self efficacy survey; MVPA: Moderate-to-vigorous physical activity; PA: Physical activity; PAR-Q: Physical activity readiness questionnaire; RCT: Randomised controlled trial; SCT: Social cognitive theory; SF-36: Short form health survey; TPB: Theory of planned behaviour; TTM: Transtheoretical model.

## Competing interests

The authors declare that they have no competing interests.

## Authors’ contributions

WKM, GSK, AJM, CV, MJD, and CMC, conceived the project and procured the project funding. GSK is leading the coordination of the trial. GSK, RRR, AJM, CV, MJD, CMC, and WKM assisted with protocol design. TNS is managing the trial including data collection with the assistance of CH. AJM and RT developed the IT platform for the trial and MJD performed the sample size calculations. GSK, RRR, and TNS drafted the manuscript and all authors read, edited, and approved the final manuscript.

## Pre-publication history

The pre-publication history for this paper can be accessed here:

http://www.biomedcentral.com/1471-2458/13/436/prepub
